# Visual cortical LFP in relation to the hippocampal theta rhythm in track running rats

**DOI:** 10.3389/fncel.2023.1144260

**Published:** 2023-06-20

**Authors:** Jack P. Kennedy, Yuchen Zhou, Yu Qin, Sarah D. Lovett, Tara Cooper, Alex Sheremet, Sara N. Burke, Andrew P. Maurer

**Affiliations:** ^1^Department of Neuroscience, College of Medicine, McKnight Brain Institute, University of Florida, Gainesville, FL, United States; ^2^Department of Psychiatry, School of Medicine, Yale University, New Haven, CT, United States; ^3^Engineering School of Sustainable Infrastructure and Environment, Herbert Wertheim College of Engineering, University of Florida, Gainesville, FL, United States; ^4^Department of Biomedical Engineering, University of Florida, Gainesville, FL, United States

**Keywords:** local field potential, hippocampus, visual cortex, volume conduction, ephaptic coupling

## Abstract

Theta oscillations in the primary visual cortex (VC) have been observed during running tasks, but the mechanism behind their generation is not well understood. Some studies have suggested that theta in the VC is locally generated, while others have proposed that it is volume conducted from the hippocampus. The present study aimed to investigate the relationship between hippocampal and VC LFP dynamics. Analysis of power spectral density revealed that LFP in the VC was similar to that in the hippocampus, but with lower overall magnitude. As running velocity increased, both the power and frequency of theta and its harmonics increased in the VC, similarly to what is observed in the hippocampus. Current source density analysis triggered to theta did not identify distinct current sources and sinks in the VC, supporting the idea that theta in the VC is conducted from the adjacent hippocampus. Phase coupling between theta, its harmonics, and gamma is a notable feature in the hippocampus, particularly in the lacunosum moleculare. While some evidence of coupling between theta and its harmonics in the VC was found, bicoherence estimates did not reveal significant phase coupling between theta and gamma. Similar results were seen in the cross-region bicoherence analysis, where theta showed strong coupling with its harmonics with increasing velocity. Thus, theta oscillations observed in the VC during running tasks are likely due to volume conduction from the hippocampus.

## Introduction

The communication between different brain areas is important for higher-order cognition, and it is thought that one way this is achieved is through the coordination of neuronal populations by low-frequency oscillations such as the 6–9 Hz theta rhythm (Buzsáki and Draguhn, 2004). The hippocampal theta rhythm, frequently recorded in rodents, becomes more prominent during spatial navigation and goal-directed tasks. However, theta activity has also been observed in various neocortical regions and has been linked to other sensory processes, such as breathing and whisking in the olfactory bulb of rodents (Moore et al., 2013; Rojas-Líbano et al., 2014; Lockmann et al., 2016; Tort et al., 2018), and saccadic eye movements in non-human primates (Jutras et al., 2013). In humans, the times between saccadic eye movements when viewing natural scenes tend to follow a normal distribution centered around theta frequency (Otero-Millan et al., 2008).

The importance of theta in sensory processes cannot be overstated, but the exact mechanisms by which it is generated across neocortical regions are still a subject of debate. It has been suggested that theta observed in cortical structures may be a result of volume conduction from high-amplitude signals generated in the hippocampus (Winson, 1974; Bland and Whishaw, 1976; Gerbrandt et al., 1978; Sirota et al., 2008; Senzai et al., 2019). However, evidence for the local generation of cortical theta has been found in the visual cortex (VC) of rats (Zold and Hussain Shuler, 2015) and in several other cortical areas in humans during working memory tasks (Raghavachari et al., 2006). Others have proposed that this activity may arise from a combination of local and volume-conducted sources (Holsheimer et al., 1979; Leung and Borst, 1987).

To further explore this issue, we used multichannel silicon probes to simultaneously record local field potentials (LFPs) from the VC and dorsal hippocampus (HPC) of rats as they traversed a circular track for a food reward. We compared three layers of the VC to the lacunosum moleculare of the HPC. While the overall magnitude of theta was lower in the cortex, all three cortical layers showed a similar increase in theta power and frequency with increases in running velocity, as observed in the hippocampus. As in previous studies, cross-frequency coupling in the hippocampus was observed between theta, its harmonics (integer, phase coupled frequencies of 7–9 Hz), and gamma (60–120 Hz). Nonlinear measures of phase coupling in the VC, however, only detected coupling between theta and its harmonics. While these observations support a volume conduction model of theta in the cortex, we offer a discussion of the potential implications of this and alternative models for activity in cortical regions when considering the role of theta in higher order cognitive processes.

## Materials and methods

### Subjects and behavioral training

**FIGURE 1:**
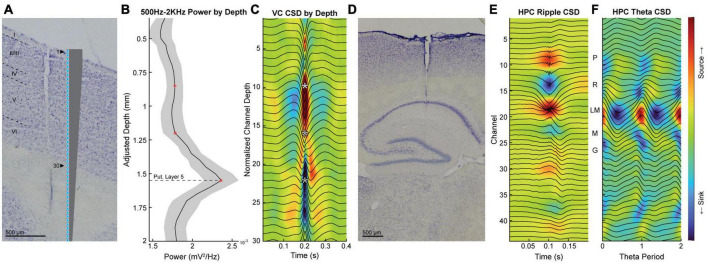
The verification of probe placement with histology and electrophysiological landmarks. **(A)** Example of histological verification of probe placement in the visual cortex with a scale diagram of the probe. **(B)** Normalized power and standard error of the mean (shaded region) within the 500 Hz to 2 KHz band in the visual cortex as a function of probe depth for all animals. Red asterisks indicate the three channels corresponding to the putative layer 5 (bottom), granule (middle), and superficial (top) layers. **(C)** Current source density triggered to high voltage spike-and-wave patterns with average LFP overlaid in black. White asterisks correspond to the locations of the red asterisks in panel **(B)**. **(D)** Histological verification of probe placement in the hippocampus. **(E,F)** Representative example of average LFP (overlaid black lines) and current source density of ripple triggered sharpwaves **(E)** and theta **(F)** used to identify hippocampal layers. PYR and LM layers are represented by dashed and dotted lines, respectively. Scale bars on histological images represent 500 μm.

Five young (4–9-months; male) Fischer 344 x Brown Norway F1 hybrid rats from the National Institute on Aging colony at Charles River were used for the current experiments. After arrival at our facilities, the rats were left undisturbed for 1 week to acclimate to the new environment. After this time, they were handled daily by experimenters. Animals were individually housed in a colony room with a reverse 12/12 light/dark cycle so that all shaping and testing procedures were carried out in the animals’ active (dark) cycle. Rats were food restricted to 85% of their *ad libitum* weight. Before implantation, rats were pre-trained to run for a food reward (45 mg, unflavored dustless precision pellets; Bio-Serv, New Jersey; Product #F0021) on a circle track 1 m in diameter (Figure 1A). After reaching a criterion of at least one lap or more per minute, rats were implanted with two linear 64-channel silicon probes manufactured by Cambridge NeuroTech (Cambridge, United Kingdom) targeting the dorsal hippocampus and primary visual cortex. Recording sites of the Cambridge NeuroTech probes were 165 μm^2^ spaced 50 μm apart, spanning 3.15 mm. Prior to surgery, probes were cleaned by soaking in a solution of 4% Contrad Detergent (Decon Contrad 70 Liquid Detergent, Fisher Scientific). Soaking was done in an oven at 55°C to accelerate the cleaning process. All procedures were conducted under the guidelines specified by the National Institute of Health Guidelines and approved by the Institutional Animal Care and Use Committee at the University of Florida.

### Surgical procedures

All surgical procedures were conducted under general isoflurane anesthesia. Rats were placed into an induction chamber and sedated with 2.5–5% isoflurane. After verifying the loss of the righting reflex, a sterile ocular ointment (Puralube Vet Ointment) was applied to prevent damage to the eyes. The top of the head was then carefully shaved, with extra care taken to avoid cutting whiskers. Rats were then secured into a stereotactic device with ear and incisor bars. After verifying a proper placement in the stereotactic device, a nose cone through which adjustable concentrations of isoflurane could be provided was secured around the snout to ensure sedation for the duration of the surgery. A piece of foil was taped to the end of the nose cone and cut to block light from shining directly into the rat’s eyes, preventing ocular damage from excessive light exposure. A non-steroidal anti-inflammatory drug (meloxicam; Boehringer Ingelheim Vetmedica, Inc., St. Joseph, MO, USA) was then administered at 5 mg/kg. Before the first incision, the site and the surrounding areas were repeatedly cleaned with alternating swabs of betadine and chlorhexidine. The primary incision began approximately just posterior to the eyes and extended slightly past the ears. Blunt dissection was used to remove the fascia attached to the skull. Bleeding was controlled via sterile saline irrigation and a battery-operated cautery pen (Bovie Medical, Clearwater, FL, USA). Stereotaxic measurements of bregma and lambda were taken, and necessary adjustments were made to ensure the skull was level. After marking lambda and bregma, the location of the craniotomy was determined and marked with the cautery tool. Following this, 7–8 anchor screws were placed around the target coordinates to serve as a foundation for the implant. Two screws were attached to copper wires that served as the ground and reference for the probe. The ground screw was placed over the cortex, contralateral to the implant, while the reference was placed over the cerebellum. A thin layer of adhesive luting cement (C&B Metabond; Parkell Inc., Brentwood, NY, USA) was then applied to form a foundation for the rest of the implant while leaving the craniotomy location uncovered. The craniotomies were completed using a 1.4 mm burr drill bit. After removing the bone, the dura was carefully retracted to avoid trauma to blood vessels and the neocortex. Saline irrigation and sterile gelatin sponges (gel foam; Pharmacia & Upjohn Co., Kalamazoo, MI, USA) were used to mitigate bleeding.

The probe was placed in a stereotaxic holder and positioned over the craniotomy at coordinates targeting the dorsal hippocampus (AP: −3.2 mm, ML: 1.5 mm, DV: −3.7 mm from the brain surface). After lowering the probe into position, the craniotomy was sealed using a bio-compatible silicone adhesive, Silastic (Kwik-Sil; World Precision Instruments, Sarasota, FL, USA). This was repeated for the second implant location targeting the visual cortex (AP: −7.1 mm, ML: 3.5 mm, DV: −3.0 mm from brain surface). Dental acrylic [Grip Cement Industrial Grade, 675571 (powder) 675572 (solvent); Dentsply Caulk, Milford, DE, USA] was used to secure the implant to the surrounding anchor screws. A small bowl of copper mesh was constructed around the implant and secured with dental acrylic to provide physical and electrostatic discharge protection. The ground and reference wires were soldered to their respective wires on both probes. The ground wires were soldered to the copper mesh, while the reference wire was electrically isolated using dental acrylic.

After verifying the stability of the implants, the rats were given 6 ml of sterile saline via subcutaneous injection, as well as a dose of buprenorphine (0.03 mg/kg; Par Pharmaceutical, Chestnut Ridge, NY, USA) given every 8 to 12 h for 24 h. After surgery, the rats were constantly observed until they were fully ambulatory and capable of eating. Meloxicam was administered twice following surgery, once 24 h after the initial dose and again at the 48-h mark. Twenty-four hours after surgery, the animals received a second dose of Metacam. No data were collected in the 7 days following surgery to allow the animals to recover while monitoring for any signs of behavioral abnormalities. During this period, the rats were also given oral antibiotics (Sulfamethoxazole/Trimethoprim Oral Suspension at 200 mg/40 mg per 5 ml; Aurobindo Pharma USA, Inc., Dayton, NJ, USA), which were mixed into their food daily.

### Neurophysiology

Following the recovery period, rats were re-run on the circle track until normal ambulatory behavior resumed, as indicated by completing at least 30 laps in 25 min. Electrophysiology data were recorded using a Tucker Davis Technologies Neurophysiology System with an acquisition rate of ∼24 kHz (PZ5, WS8, RV2, and RZ2, Alachua, FL, USA). Position data were recorded by tracking red and green LEDs on the headstage at ∼30 fps with a 0.27 cm/pixel resolution. Each recording session consisted of a 25-min run between two 25-min periods of rest in a ceramic flowerpot (Figure 1C).

### Analyses and statistics

All analyses were performed in MATLAB^®^ (MathWorks, Natick, MA) using custom-written code as well as code imported from the HOSA toolbox (Swami et al., 2003). Raw neurophysiology data were downsampled from ∼24 kHz to ∼2 kHz and divided into 1 s LFP segments. Segments with values that exceeded 10 or more standard deviations from the mean were excluded from analysis. The analysis methods used in the current experiments have previously been described in detail (Sheremet et al., 2016, 2019; Zhou et al., 2022) and are based on standard techniques for spectral and time series analysis (Priestley, 1981; Papoulis and Pillai, 2002). Position data was obtained by using a camera to track LEDs attached to the headstage at 30 FPS. Pixels were converted into centimeters, and any gaps in position data were interpolated using the MATLAB function “INPAINTN” (Garcia, 2010). Initial smoothing of position data was accomplished by convolving x and y points with a Gaussian window of 0.5 s. The velocity of each rat was calculated by dividing the linear distance between adjacent points by the difference between timestamps associated with those points. Velocity was interpolated to match the ∼2 kHz sampling rate of the LFP data. Non-functional channels were excluded from all analyses with the exception of the current source density analyses (CSD) in Figure 1, where they were replaced by the linear interpolation of adjacent channels. These substitutions did not impact the layer channels chosen for further analysis. Traditional current source density analysis was used to identify the stratum pyramidale, stratum radiatum, and stratum lacunosum moleculare of the hippocampus (Rappelsberger et al., 1981; Mitzdorf, 1985; Buzsáki et al., 1986; Bragin et al., 1995). To calculate theta CSDs, LFP segments were filtered between 4 and 30 Hz and aligned to theta peaks on the channel with the highest theta power. The second spatial derivative for each segment was calculated prior to averaging across all segments to obtain the average theta CSD. The same was done for ripple CSDs with the exception that the LFP was not filtered and aligned to the maximum ripple peak in the pyramidal layer [please see Figure 1 in Sheremet et al. (2019)]. The visual cortex used a similar approach, triggering the CSD to high voltage spike-and-wave events (Kandel and Buzsáki, 1997). Depth adjustment was performed to provide a rough estimate of cortical electrode location by aligning features of the individual 500 Hz to 2 kHz power-by-depth plots and using electrode site spacing to estimate site depth in a manner similar to that of Senzai et al. (2019).

### Database

Four datasets from four mice previously published in Senzai et al. (2019) were generously provided by the Buzsaki Lab and obtained from the public webshare on the Buzsaki Lab website. The raw LFP data was downsampled to ∼2 kHz and processed as described above. Position tracking information was not available for these animals and, as such, only velocity independent analyses were conducted.

## Results

To accurately determine the locations of our electrodes, we verified placement using current source density analysis and histology. Figure 1 illustrates the localization of electrodes in the visual cortex (A-C) and hippocampus (D-F). In a similar manner to Figure 1 of Senzai et al. (2019), we identified the putative cortical layers of the visual cortex using average LFP power by depth in the 500–2 kHz range (Figure 1B) and current source density analysis triggered to spontaneously evoked activity (Figure 1C; Schaefer et al., 2015). The layers of the hippocampus were determined by identifying current sources and sinks using current source density analysis triggered to ripples to identify the pyramidal layer (Figure 1E) and theta to identify the lacunosum moleculare layer (Figure 1F; Berényi et al., 2014).

**FIGURE 2:**
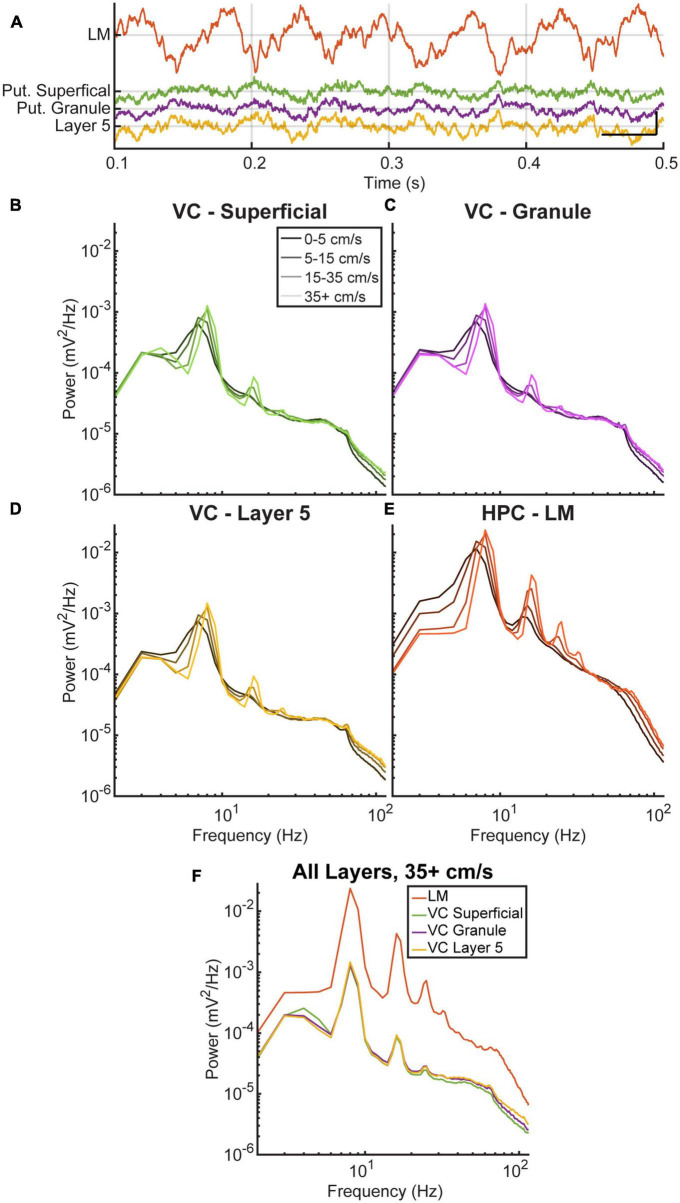
Theta and average power spectral density in the visual cortex and hippocampus. **(A)** A qualitative example comparing 1 s of theta in the hippocampus (PYR, RAD, LM, in black) to theta in the visual cortex (colored) during running. Vertical and horizontal scale bars represent 0.5 mV and 100 ms, respectively. **(B–E)** Average power spectral density for three layers of the visual cortex **(B–D)** and one layer of the hippocampus **(E)** as a function of running velocity. Each colored line corresponds to a different velocity bin. All layers show a similar increase in the power and frequency of theta and its harmonics with increasing velocity. **(F)** Comparison of the four layers at the highest velocity bin. The cortical layers analyzed show a similar pattern in the increase of theta power and harmonics with increasing velocity.

There is ongoing debate about whether the cortical theta rhythm is generated locally or is the result of volume conduction (Petsche et al., 1960). To investigate this further, we analyzed the power spectra of three cortical layers and the lacunosum molecular of the hippocampus across different running velocities. Figure 2A shows an example of the raw LFP data during a period of high running velocity, where theta is visibly present in the cortical data at a lower amplitude compared to the hippocampus. A repeated-measures two-factor ANOVA was run with Tukey’s multiple comparisons tests comparing theta power across all four locations of interest and velocities. The ANOVA indicated a main effect of location [*F*_(1.0005,4.0019)_ = 30.0, *p* = 0.005] and velocity [*F*_(1.2645,5.0581)_ = 26.2, *p* = 0.003] as well as an interaction effect between the two [*F*_(1.1999,4.7998)_ = 17.0, *p* = 0.009]. In all areas of interest, there was a significant increase in theta power at the highest velocity, >35 cm/s, compared to the low velocity conditions of 0–5 cm/s [*t*_(20)_ = −5.46, *p* = 0.0001] and 5–15 cm/s [*t*_(20)_ = −4.15, *p* = 0.003], illustrated Figures 2B–E. As is evident in Figure 2F, peak theta power in the lacunosum-moleculare is greater than all cortical layers (Tukey’s multiple comparison, VC-L5 *p* < 0.0001, 95% CI = [0.011, 0.024]; VC-Gran *p* < 0.0001, 95% CI = [0.011, 0.024]; VC-Sup *p* < 0.0001, 95% CI = [0.011, 0.024]). Despite the lower overall power, the power spectra of the three visual cortical layers exhibited changes in the power and frequency of theta and its harmonics with increasing velocity, similar to what was observed in the hippocampus. At the highest velocities (>35 cm/s), theta power in VC-L5 was greater than VC-Gran (*p* = 0.034, 95% CI = [0.00001, 0.0002]) but not VC-Sup (*p* = 0.126, 95% CI = [−0.00008, 0.0005]). Additionally, there was no difference in theta power between VC-Gran and VC-Sup (*p* = 0.58, 95% CI = [−0.0002 to 0.0004]) (Figure 2F).

**FIGURE 3:**
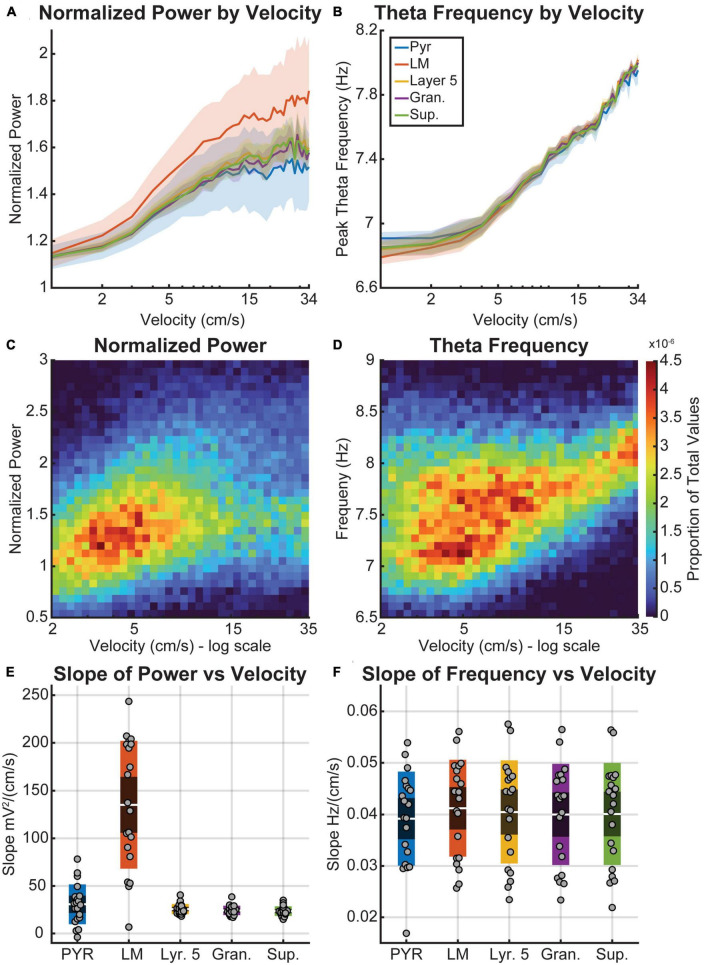
Theta power and frequency as a function of velocity. **(A,B)** Semi-log plots of the average normalized theta power **(A)** and frequency **(B)** by velocity for each layer (colored lines). The shaded region represents the standard error of the mean between the five animals. **(C,D)** 2-Dimensional histogram plots of theta power **(C)** and frequency **(D)** by velocity. Warmer colors indicate bins with a higher proportion of the total values. Note that the x-axis of plots **(C,D)** is logarithmically scaled. **(E,F)** Box plots average data overlaid as points showing the average slope of the best-fit line for theta power **(E)** and frequency **(F)** by velocity for each layer. The red bands indicate a 95% confidence interval around the mean (central red line), with one standard deviation indicated by the blue bands on either side. Theta power was normalized using the y-intercept of the line fit to the theta power by velocity plots.

To further examine the changes in power and frequency relationships, we analyzed the rate of change between velocity and theta power/frequency as a function of layer (Figure 3). Shaded error bar plots show the averages of normalized theta power (Figure 3A) and theta frequency (Figure 3B) by velocity. Theta power was normalized by the y-intercept determined from a robust multilinear regression of the theta power-velocity plots (MATLAB function “robustfit.m”), which can be thought of as the hypothetical theta power at 0 cm/s. While the average normalized theta power was still larger in the hippocampus LM (*Post hoc* Comparisons with Tukey correction: VC-L5 *t*_(679)_ = 5.3, *p* < 0.0001; VC-Gran *t*_(679)_ = 6.1, *p* < 0.0001; VC-Sup *t*_(679)_ = 5.6, *p* < 0.0001), all three cortical layers of the visual cortex showed a similar trend of increasing power with velocity [*F*_(33,679)_ = 6.3, *p* < 0.0001]. Data for the lacunosum moleculare is shown as a 2D histogram for normalized power (Figure 3C) and frequency (Figure 3D) to illustrate the overall distribution of values as a function of velocity. The slopes obtained from these data provide a measure of velocity sensitivity of power or frequency within a region, indicating how much theta power changes with increasing velocity. To determine the distribution of slopes, we compared the effect of velocity on power and frequency in each region (see box plots 3E/F). The average slope of power versus velocity was significantly higher in the hippocampus than in the cortical layers [*t*_(679)_ = 5.298, *p* < 0.0001]. There was no significant difference in the average power-velocity slope values among the layers of the visual cortex (*p* > 0.95). In contrast to power, the frequency-velocity slopes showed no significant difference across any layers (*p* = 0.88, CI = [−0.01, 0.0098]).

**FIGURE 4:**
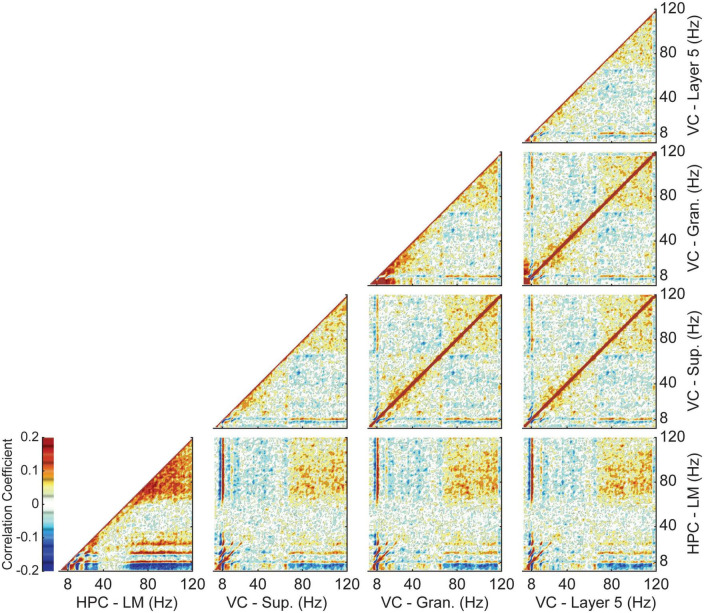
Average Power-power correlations between the hippocampus and visual cortex. Each column and row correspond to a different layer. Panels along the diagonal show the autocorrelation of that layer. Dark red colors indicate areas of strong positive correlation, while blue indicates a more negative correlation. Theta power in the visual cortex shows a strong correlation with both theta and gamma power in the lacunosum moleculare of the hippocampus.

As previously shown, theta power in the visual cortex is significantly lower than in the hippocampus. However, normalization via the y-intercept of a multilinear regression brings the two regions closer in terms of the relative changes in theta power with velocity, indicating a consistent increase in power with velocity regardless of region. To examine the relationship between theta power in the two regions independently of velocity, we performed a power-power correlation analysis within and between all four regions of interest. The results of which were averaged across all animals and are shown in Figure 4. The method chosen is advantageous as it enables a comparison across frequencies without the need for filtering (Buzsáki et al., 2003; Masimore et al., 2004, 2005). For all pairwise cases, theta power in one region was highly correlated with theta power in the other. Similarly, auto-power correlations for each layer showed a high correlation between theta and gamma (60–120 Hz). Power correlations between layers of the visual cortex were largely symmetrical, with theta power in one region being correlated with theta and gamma power in the other and vice versa. The main differences occurred when comparing layers of the visual cortex to the lacunosum moleculare. Visual cortex theta power was consistently and strongly correlated with gamma power in the lacunosum moleculare, while LM theta power showed a much weaker correlation with VC gamma power. The level of correlation between LM theta power and VC gamma power was similar to that seen between the different layers of the visual cortex.

**FIGURE 5:**
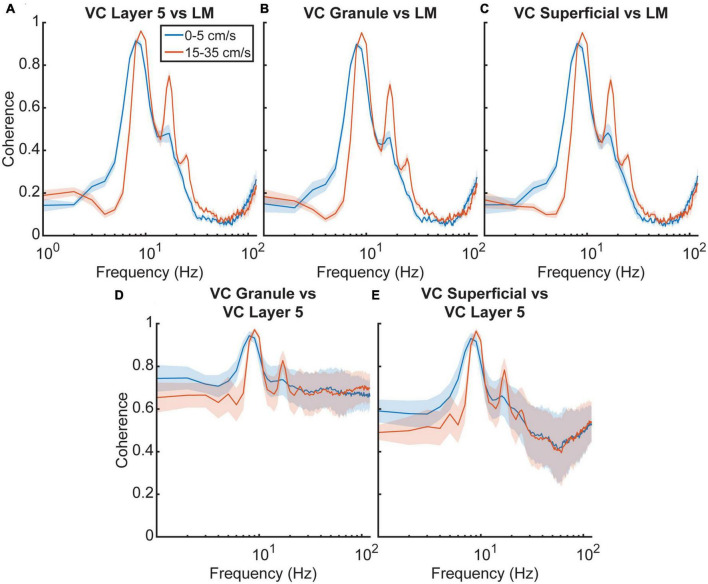
Cross power spectral density of hippocampus and visual cortex. **(A–C)** The cross power spectral density (CPSD) between three visual cortex layers and the lacunosum moleculare of the hippocampus as well as the putative granule for five animals as a function of velocity. **(D,E)** CPSD between putative visual cortex layer five and the two remaining visual cortex layers. Dark lines correspond to the average value, with blue corresponding to low velocities and orange corresponding to high velocities. The shaded regions correspond to the standard error of the mean. In all instances, the CPSD shows a slight increase at theta frequency and a more significant increase in the theta harmonics with velocity.

**FIGURE 6:**
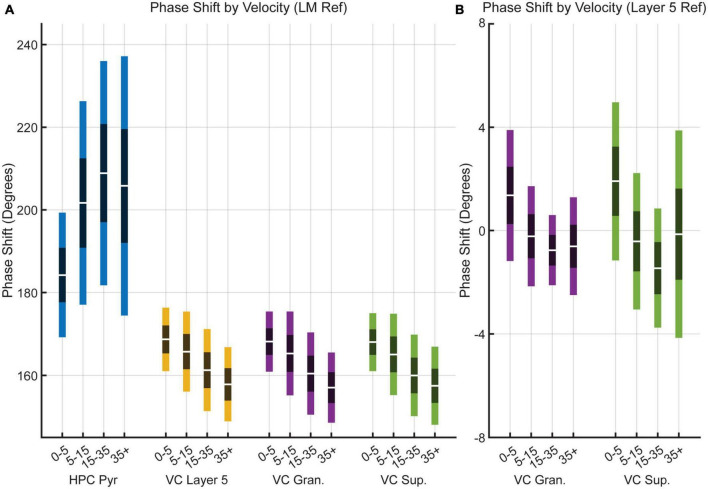
Theta phase shift as a function of velocity. **(A)** Box plots showing the change in theta phase in the HPC pyramidal (blue), VC Layer 5 (orange), VC granule (yellow), and VC superficial (purple) over four velocity bins relative to the lacunosum moleculare of the hippocampus. **(B)** Box plots showing theta phase change relative to putative visual cortex layer 5 in the granule (yellow) and superficial (purple) layers of the visual cortex. Note the significantly smaller scale on the y-axis compared to panel **(A)**. Dark bands indicate a 95% confidence interval around the mean (central white line) with one standard deviation indicated by lighter bands on either side.

To examine the frequency relationship between the VC and the LM further, we analyzed cross power spectral densities to illustrate changes in coherence between the different regions (Figure 5). A repeated-measures two-factor ANOVA with velocity and region of interest as factors indicated that there was a main effect of velocity [*F*_(3,12)_ = 22.9, *p* = 0.00003] but not region [*F*_(3,12)_ = 0.6, *p* = 0.6] on theta coherence between the HPC and VC, suggesting that there is a consistent increase in theta coherence across regions as velocity increases, which is visualized in Figures 5A–C. Looking at theta coherence within layers of the visual cortex by using VC Layer 5 as a reference, there is again a main effect of velocity [*F*_(3,12)_ = 14.0, *p* = 0.0003] but not region [*F*_(3,12)_ = 2.6, *p* = 0.1], however, there is a significant interaction effect between the two [*F*_(3,12)_ = 7.0, *p* < 0.00001] which can be seen in Figures 5D, E. These results tentatively suggest that theta seen in the visual cortex arises from volume conduction from the hippocampus. If there were individual generators of theta between the VC and the HPC, one could imagine relative variability in phase offsets across regions. Figure 6 shows the results of phase offset calculations for different velocity bins using the lacunosum moleculare (Figure 6A) and putative VC layer 5 (Figure 6B) as references. Using LM theta as a reference, the average theta phase shift in the VC was around 168° at the lowest velocity bin, decreasing to around 157° at velocities greater than 35 cm/s. These results showed a marked consistency across the three layers of the VC across all velocity bins. For comparison, the phase shift in the pyramidal layer of the hippocampus (HPC) is also shown, as it represents a layer with a well-defined phase offset compared to the lacunosum moleculare. Within the visual cortex, there was little phase shift (<±2° on average) between putative layer 5 and the putative superficial or granule layers. *Post hoc* analyses with Tukey corrections support this statement, indicating the phase shift between visual cortex putative layer 5 and the lacunosum moleculare is greater than that with either the granule layer [*t*_(16)_ = 149.766, *p* < 0.00001] or superficial layer [*t*_(16)_ = 168.598, *p* < 0.00001] of the visual cortex. Furthermore, the phase shift between VC layer 5 and VC granule cells layer does not differ from that with VC superficial layers [*t*_(16)_ = −0.113, *p* = 0.999].

**FIGURE 7:**
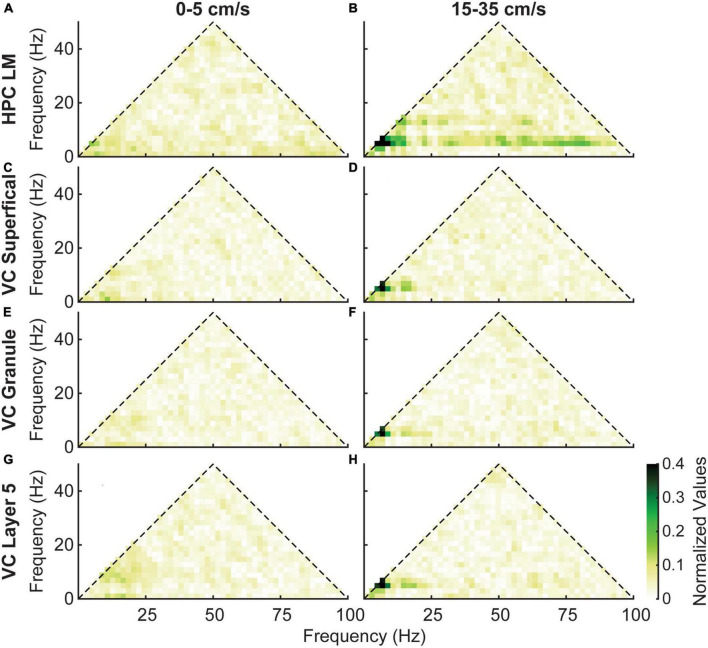
Average bicoherence estimates in the lacunosum moleculare **(A,B)** and three layers of the visual cortex **(C–H)** for low (5–0 cm/s, left column) and high (15–35 cm/s, right column) velocities. Layers of the visual cortex show a similar evolution of coupling between theta and its harmonics with increasing velocity. However, there is a distinct lack of coupling between theta and gamma in the visual cortex.

Previous studies have demonstrated that in the hippocampus, phase coupling between theta oscillations, their harmonics, and gamma oscillations increases with velocity (Chen et al., 2011; Ahmed and Mehta, 2012; Sheremet et al., 2018, 2019; Zhou et al., 2022). To examine this phenomenon in the visual cortex, we performed bicoherence analyses. At low velocities (as shown in Figure 7, left column), phase coupling between theta and its harmonics was minimal in both the hippocampus and visual cortex. However, at high velocities (Figure 7, right column), we observed an increase in phase coupling between theta and its harmonics in all regions. In comparison to the hippocampus, the VC showed a lack of phase coupling between theta and gamma oscillations.

**FIGURE 8:**
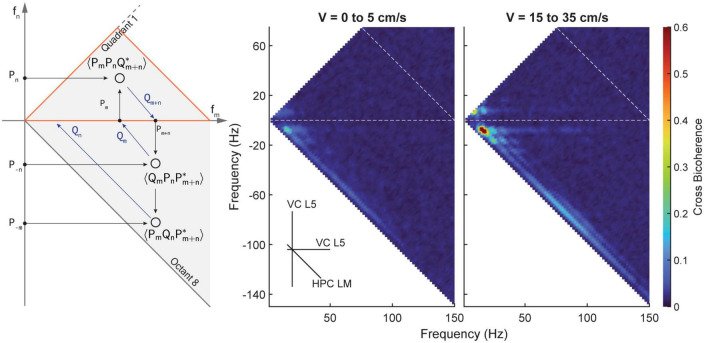
Average cross bicoherence estimates between the lacunosum moleculare and layer 5 of the visual cortex for low (5–0 cm/s, middle) and high (15–35 cm/s, right) velocities. Layers of the visual cortex show a similar evolution of coupling between theta and its harmonics with increasing velocity. However, there is a distinct lack of coupling between theta and gamma in the visual cortex.

**FIGURE 9:**
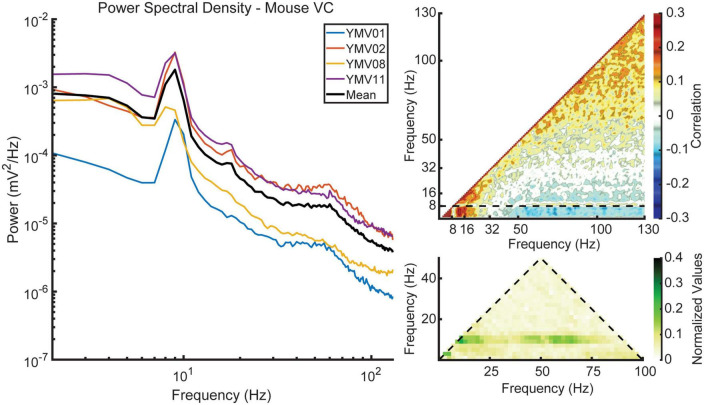
Data obtained from mice courtesy of Senzai et al. (2019). All three measures show similar trends to those seen in the data collected from rats, with the exception of strong coupling between theta and gamma phase, as seen in the bicoherence plot.

To investigate the existence of any cross-region phase coupling between the hippocampus and visual cortex, we conducted cross-bicoherence analyses. Figure 8 presents the coupling between the putative layer 5 of the visual cortex (depicted on the two primary axes) and the lacunosum moleculare of the hippocampus (depicted on the third, diagonal axis). These analyses reveal changes in coupling strength with respect to velocity, similar to what was observed in the standard bicoherence analyses. To further strengthen the validity of our results, we also included a cross-species comparison of visual cortex data from mice in our study. Figure 9 shows the average power spectral density, power-power correlation, and bicoherence analyses of the mouse visual cortex, based on data from Senzai et al. (2019). Although velocity data was not available for these mice, the velocity-independent results closely resembled those obtained in our study. One notable exception was the presence of theta-gamma coupling in the mice bicoherence plot (Figure 9C), which may be due to the shorter distance over which volume conduction must occur in mice compared to rats.

## Discussion

The present study aimed to investigate the relationship between hippocampal and visual cortical (VC) LFP. Analysis of power spectral density revealed that LFP found in the VC was similar to that in the hippocampus (HPC), but with lower overall magnitude. As velocity increased, both the power and frequency of theta and its harmonics increased in the VC (Figures 2, 3). However, this increase in cortical theta frequency was not significantly different from what was observed in the HPC (Figure 3) [*F*_(4,20)_ = 0.0309, *p* = 0.999]. Current source density analysis triggered to theta did not identify distinct current sources and sinks in the cortex, supporting the idea that theta in the VC is conducted from the adjacent HPC.

To this effect, we found that VC theta power was consistently coupled to both theta and gamma power in the HPC, with the reverse holding true as well (Figure 4). In the same vein, coherence at theta frequency between the different layers of VC and the HPC lacunosum moleculare was high, with further increases in coherence with faster running velocity (Figure 5). Comparison of the phase shift between the HPC and VC was found to be close to 180 degrees in all cortical layers, and consistently decreased with velocity (Figure 6). The marked phase difference between cortical and hippocampal theta could be due to the distance between the two recording electrodes and the observation that hippocampal theta has been shown to travel along the dorsal-ventral axis (Lubenov and Siapas, 2009).

Phase coupling between theta, its harmonics, and gamma is a notable feature in the HPC, particularly in the lacunosum moleculare. While some evidence of coupling between theta and its harmonics in the VC was found, bicoherence estimates did not reveal significant phase coupling between theta and gamma (Figure 7). Similar results were seen in the cross-region bicoherence analysis, where theta showed strong coupling with its harmonics with increasing velocity (Figure 8). Many of the results seen here were recapitulated in the mouse data provided by the Buzsaki laboratory (Figure 9). The most notable exception being the prominent phase coupling between theta and gamma in the mouse visual cortex (Figure 9, bottom right panel). Due to the differences in brain size between rats and mice, it is conceivable that volume conduction of hippocampal gamma may be the reason for this discrepancy.

The present study provides evidence that theta oscillations observed in the VC during running tasks are likely due to volume conduction from the hippocampus. This conclusion was supported by the similarity of theta power and frequency dynamics in the VC and HPC, as well as the lack of distinct current sources and sinks in the VC, as determined by current source density (CSD) analysis triggered to theta oscillations. It should be noted that these findings were obtained from animals running under relatively dark conditions. Other studies have demonstrated that visual stimulation can elicit locally generated theta oscillations in the VC (Zold and Hussain Shuler, 2015). In contrast, a study by Torres et al. (2019) found that, under urethane anesthesia, a significant portion of LFP activity in the VC can be attributed to volume conduction from the HPC. Similar observations of volume conduction have also been made in other brain regions, such as the dorsolateral striatum, where theta oscillations during running tasks are thought to arise from volume conduction from the hippocampus (Lalla et al., 2017). However, much like the reports of both volume-conducted and locally generated theta in the cortex, evidence supporting the local generation of theta found in the striatum has also been found (DeCoteau et al., 2007), indicating that the observed LFP may be a combination of multiple factors.

Traditionally, “volume conduction” is described as the electric potential measured some distance away from the point of origin; the process by which electrical activity from an active neuron’s membrane potential spreads through the surrounding tissue. It is a passive and resistive process, meaning that the strength of the electrical signal decreases as the distance from the active membrane increases (Holsheimer and Feenstra, 1977; Blum and Rutkove, 2007; Lindén et al., 2010; Buzsáki et al., 2012).

There have been several models proposed to explain the generation of theta rhythms in the cortex. One model suggests that the theta seen in the cortex is locally generated, as indicated by the presence of sources and sinks across cortical layers in rats (Zold and Hussain Shuler, 2015). Additionally, Raghavachari et al. (2006) found a lack of theta coherence across different cortical sites, while there was high coherence between depth and surface electrodes in humans. These observations would suggest that the theta seen in the cortex, of humans at least, is generated at different locations.

Another model suggests that the theta seen in the cortex is generated via volume conduction from the HPC or other regions such as the medial entorhinal cortex. Sirota et al. (2008) found that neocortical theta frequency and amplitude covaried with hippocampal theta and that prefrontal cortex unit activity was more coherent with hippocampal LFP compared to locally recorded LFP. Senzai et al. (2019) also suggest that the theta seen in the visual cortex is volume conducted from the HPC. This perspective is in general agreement with the prior history of the literature at large (Holsheimer and Feenstra, 1977).

A third model suggests that the generation of theta in the cortex may involve a combination of local generation and volume conduction. Holsheimer (1982) found a high degree of coherence between cingulate cortex and hippocampus theta, with a phase reversal between the two (also see Feenstra and Holsheimer, 1979). They also found that cortical units fired in phase with theta in the cingulate cortex and proposed that this was due to the influence of the medial septal-diagonal band of Broca, a structure known to be important for the generation of theta in the hippocampus (Swanson and Cowan, 1979). However, Leung and Borst (1987) found that cingulate cortex theta was phase-locked to HPC theta and likely the result of volume conduction but did not observe the phase reversal described by Holsheimer. They also found that septal lesions, which eliminated HPC theta, did not affect cingulate cortex theta in some animals, suggesting that the cingulate cortex has the capability for theta generation. Kajikawa and Schroeder (2011) showed that LFP can be detected at several millimeters away from the site of generation, regardless of frequency content, which can impact areas where locally generated LFP is relatively weak or disorganized. These weaker sources can be “contaminated” by the spread from a stronger source, which lends support to a large reentrant circuit model of theta generation (Buzsáki and Draguhn, 2004).

A significant limitation of the present study is that it did not isolate single unit activity in either the VC or HPC. If single unit action potentials were modulated by hippocampal theta, it would suggest that if the LFP is primarily volume conducted from the HPC to the cortex, the cortical neurons are somehow sensitive to this. A previous study from the mouse VC found that approximately one quarter of visual cortical neurons are modulated by theta (Fournier et al., 2020). Similarly, several studies have found evidence of neuronal modulation of cells by the hippocampal theta rhythm in the prefrontal cortex (Siapas et al., 2005), neocortex (Sirota et al., 2008), and medial and lateral entorhinal cortices (Deshmukh et al., 2010). Entertaining the idea that visual cortical entrainment to hippocampal theta is due to volume conduction, theta oscillations transmitted from the HPC to the VC may be influencing the activity of principal neurons in the cortex directly or activating interneurons which in turn modulate the activity of principal neurons. That is, volume conducted theta may have a physiological influence in synchronizing neuronal populations via ephaptic coupling (Zhang et al., 2014; Anastassiou and Koch, 2015; Rebollo et al., 2021; Subramanian et al., 2022). Regarding the lack of single neuronal recordings in our study, inhibitory modulation may also contribute to the theta-phase-related firing of units in the visual cortex. Inhibitory modulation may occur without large CSDs in the visual cortex, because of the lack of sensitivity of CSDs to detect synchronous inhibitory potentials. This may potentially explain the difference between the cross-frequency theta-gamma coupling in the Yuta et al. study in mice (Figure 9) and the rat data presented here (Figure 7).

Although ephaptic coupling initially implied a contact-based coupling, over time, it has perhaps become somewhat of an etymologic misnomer, evolving to include field effects and volume conduction. Non-synaptic methods of communication, such as field effects, have been studied since the 1940s (Katz and Schmitt, 1940). It is well established that electric fields can exert an influence on neuronal activity, impacting function at multiple scales. On the level of the individual neuron, small electric fields produce changes in neuronal excitability (Bikson et al., 2004), and when paired with concurrent synaptic input, can modulate the spike timing and coherence of CA1 pyramidal neurons (Radman et al., 2007). On a network level, electric fields with naturalistic amplitudes modulate neocortical activity, enabling a feedback mechanism between the electric field and the neurons that give rise to it (Fröhlich and McCormick, 2010). Previously, it was believed that the influence of ephaptic coupling was limited to events where large amounts of synchronous activity occur, such as during epilepsy. More recent studies have shown that the effects of ephaptic coupling are capable of influencing adjacent regions during normal levels of electrophysiological activity (Rebollo et al., 2021). Changes to extracellular voltages through volume conduction from spatially close but synaptically distant regions will influence membrane potentials and therefore firing probabilities (Anastassiou and Koch, 2015; Subramanian et al., 2022). Furthermore, the almost instantaneous nature of electric fields could enable synchronization between areas faster than is possible via synaptic connections alone. While the focus thus far has been on role of electric fields in mediating activity between brain regions, it is important to note that this is not the only method by which oscillatory activity may propagate. Waves of spiking activity have been shown to propagate in cortical regions (Bringuier et al., 1999) and a model for the transfer of information via spiking activity propagation has been put forth (Kumar et al., 2010). Interactions such as these have substantial implications concerning brain states and behavior and warrant further investigation into mechanisms not traditionally considered when investigating cross-region communication.

However, a model of recurrently connected brain regions, roughly approximating a ring, could also account for the wide reports of theta in multiple brain regions (Skaggs, 1995; Vertes et al., 2001; Buzsáki and Draguhn, 2004). As offered by Fournier et al. (2020), there may exist a HPC-retrosplenial-visual cortex loop, in which synaptic conduction across regions determines the frequency of theta and the number of neurons entrained determine the amplitude. Alternatively, the HPC and the VC receive common inputs from the medial septum, providing a different mechanism of coordination across regions (Carey and Rieck, 1987; Gaykema et al., 1990; Unal et al., 2015). Therefore, it is possible that the septal-hippocampal feedback loop (Cheney and Panula, 1986; Witter et al., 2000) coordinates the rhythmicity of neurons in the medial septum that project to the cortex.

It is tenable that the actual mechanism resides between these two extremes. In the lateral habenula, whose neuronal populations lack an organized structure, the majority of LFP measured was found to be the result of the much “louder” HPC drowning out what endogenously generated LFP was present (Bertone-Cueto et al., 2020). Should a similar phenomenon occur in the VC, the LFP is primarily a consequence of volume conduction while the neuronal entrainment of VC neurons to theta is supported via common anatomical networks. As evidenced by Senzai et al. (2019), cortical gamma is a distinct feature of the cortex, enough so that it can be used in conjunction with local spiking activity to determine anatomical layers. In the present study, we show that there is strong coupling between theta and gamma seen in the cortex. This coupling would indicate that the pyramidal-interneuron microcircuits responsible for the generation of gamma in the visual cortex are entrained to a larger theta rhythm, regardless of its origin. Extending this idea one step further, it is conceivable that the ionic flux in the LFP and the local synaptic activity may operate in a subtle, yet synergistic, manner to support neural coordination between different brain regions.

## Data availability statement

The raw data supporting the conclusions of this article will be made available by the authors, without undue reservation.

## Ethics statement

The animal study was reviewed and approved by the University of Florida Institute of Animal Care and Use Committee.

## Author contributions

AM and SB contributed to the conception and design of the study. SL and JK performed the research. JK, YZ, YQ, and AS provided analytical tools. JK conducted the data analysis and generated the figures. TC conducted statistical analyses. JK and AM wrote the first draft. All authors contributed to the manuscript revision and read and approved the submitted version.
